# Mechanically Spatio‐Chimeric Fibrin Assembly Enables Vascular‐Integrated Muscle Reconstruction for Volumetric Muscle Loss Repair

**DOI:** 10.1002/adma.202523542

**Published:** 2026-04-22

**Authors:** Su Hyun Jung, Minjun Kim, Da‐Yoon Kim, Min Kyu Kim, Sieun Lee, Yoonhee Jin, Joo H. Kang

**Affiliations:** ^1^ Department of Biomedical Engineering College of Information and Biotechnology Ulsan National Institute of Science and Technology (UNIST) Ulsan Republic of Korea; ^2^ Department of Physiology Graduate School of Medical Science Brain Korea 21 Project Yonsei University College of Medicine Seoul Republic of Korea

**Keywords:** anisotropic 3D muscle scaffolds, mechanically bimodal hydrogel systems, shear‐driven fibrin assembly, vascular‐integrated muscle graft, volumetric muscle loss repair

## Abstract

Volumetric muscle loss (VML), a severe injury involving irreversible loss of both muscle tissue and vasculature, poses a major barrier to the development of clinically viable muscle grafts. Functional restoration requires engineered constructs capable of reconstructing both contractile and vascular components that can functionally integrate with the host vasculature. Here, we introduce SPARC (spatio‐chimeric, plasma‐based, anisotropic, and shear‐responsive construct), a mechanically bimodal fibrin hydrogel engineered via shear‐guided assembly of plasma fibrin to recreate the structural and mechanical heterogeneity of native muscle. Controlled microfluidic shear generates aligned fibrillar bundles and a spatially graded bimodal stiffness architecture, establishing stiff, bundle‐dense regions that favor myogenic differentiation and compliant regions that promote endothelial morphogenesis. When co‐cultured with myoblasts and endothelial cells, the resulting anisotropic matrix directs spatially organized myogenic maturation and endothelial morphogenesis. In vivo evaluation in a murine VML model shows that vascularized muscle SPARC grafts restore muscle architecture and function, promoting neovascularization, myofiber regeneration, and enhanced motor recovery. Through its spatially mechano‐programmed design, SPARC enables coordinated myogenic and endothelial organization within a single construct, establishing a scalable biofabrication strategy for functional repair of extensive muscle defects.

## Graphical abstract

SPARC (spatio‐chimeric, plasma‐based, anisotropic, and shear‐responsive construct) that integrates myogenic and vascular microenvironments within a single construct. The dual‐modulus matrix directs aligned myotube formation and endothelial network development, enabling a vascularized muscle implant that seamlessly anastomoses with host tissue and restores structural and functional integrity after volumetric muscle loss.

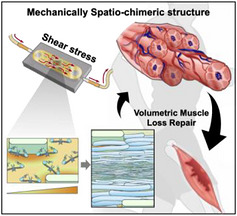

## 1 Introduction

Engineering physiologically relevant tissue constructs remains a persistent challenge in regenerative medicine and disease modeling. Native tissues such as skeletal muscle and vasculature exhibit integrated spatial heterogeneity, where anisotropic architectures and tissue‐specific mechanical cues together simultaneously drive myogenic and vascular differentiation while preserving their respective functions. These tissues comprise multicellular compartments that differ in stiffness, biochemical signaling, and the orientational order of the local microenvironment, yet remain interconnected within a continuous extracellular matrix (ECM). Replicating such integrated heterogeneity in vitro is essential for constructing functional grafts. However, current hydrogel systems, including collagen, Matrigel, or synthetic polymers like PLGA, fail to reflect the compositional and mechanical diversity of native tissues [1, 2, 3]. While modular assembly approaches, such as stacking or mixing hydrogels of differing composition, have achieved some spatial variation, they typically lack mechanical continuity and disrupt matrix‐integration across interfaces [4].

In addition to stiffness variation within grafts, anisotropic topological cues of the ECM are also critical, particularly in mechanically active tissues such as skeletal muscle, where they organize contractile myofibers and contribute to the spatial organization of endothelial structures, guiding myoblast fusion and endothelial morphogenesis [5, 6, 7]. Although several methods have been developed to impose their alignment, these strategies often fail to concurrently recapitulate the stiffness requirements of distinct tissues or maintain structural integration across adjacent regions [8]. Another key challenge is combining aligned ECM architecture with region‐specific mechanical properties in a single engineered matrix. Endothelial and myogenic lineages exhibit distinct stiffness preferences; vascular morphogenesis occurs optimally in soft, compliant environments (11–250 Pa), whereas myogenic differentiation requires significantly stiffer substrates (10–50 kPa) [5, 9, 10, 11, 12]. Yet many currently available scaffolds present either isotropic topographies or uniform stiffness, rarely accommodating both cues in a coordinated manner. Moreover, the absence of endogenous biochemical signals, such as myokines or growth factors, further limits their capacity to support lineage‐specific maturation. As a result, most current platforms face limitations in recapitulating the integrated microenvironment required for the concurrent differentiation and spatial organization of multiple cell types within a single construct.

Here, we present a biofabrication strategy to generate a heteromechanical hydrogel system, termed SPARC (spatio‐chimeric, plasma‐based, anisotropic, and shear‐responsive construct) that captures key mechanical and biochemical features of native vascularized skeletal muscle tissues. By generating a spatial gradient of fluidic shear stress when blood plasma flows through a microfluidic channel, fibrin fibers self‐assemble into aligned bundles with locally tunable bundle diameter and packing density, yielding a monolithic scaffold that concurrently spans the elastic modulus ranges of muscle and vascular tissues. Mechanistically, we show that directional fibrin bundle formation under shear flow is mediated by electrostatic interactions between fibers, which facilitate bundling and guide anisotropic fiber assembly. The SPARC platform reconstructs the architectural, mechanical, and biochemical key features of native tissues within a single monolithic construct. In vitro, it supported parallel myotube alignment, vascular network formation, and spatially coordinated maturation. Upon implantation into a rodent model of extensive volumetric muscle loss (VML), the constructs integrated with host tissues, promoted neovascularization accompanied by vascular anastomosis, restored muscle architectures, and significantly improved motor recovery. By unifying multi‐modulus mechanics, fibrillar anisotropy, and intrinsic ECM‐mimetic cues, SPARC establishes a clinically scalable strategy for muscle reconstruction with vascular integration.

## 2 Results and Discussion

### 2.1 Shear‐Proportional Fibrin Bundling Enables Anisotropic, Mechanically Heterogeneous 3D Constructs

**FIGURE 1:**
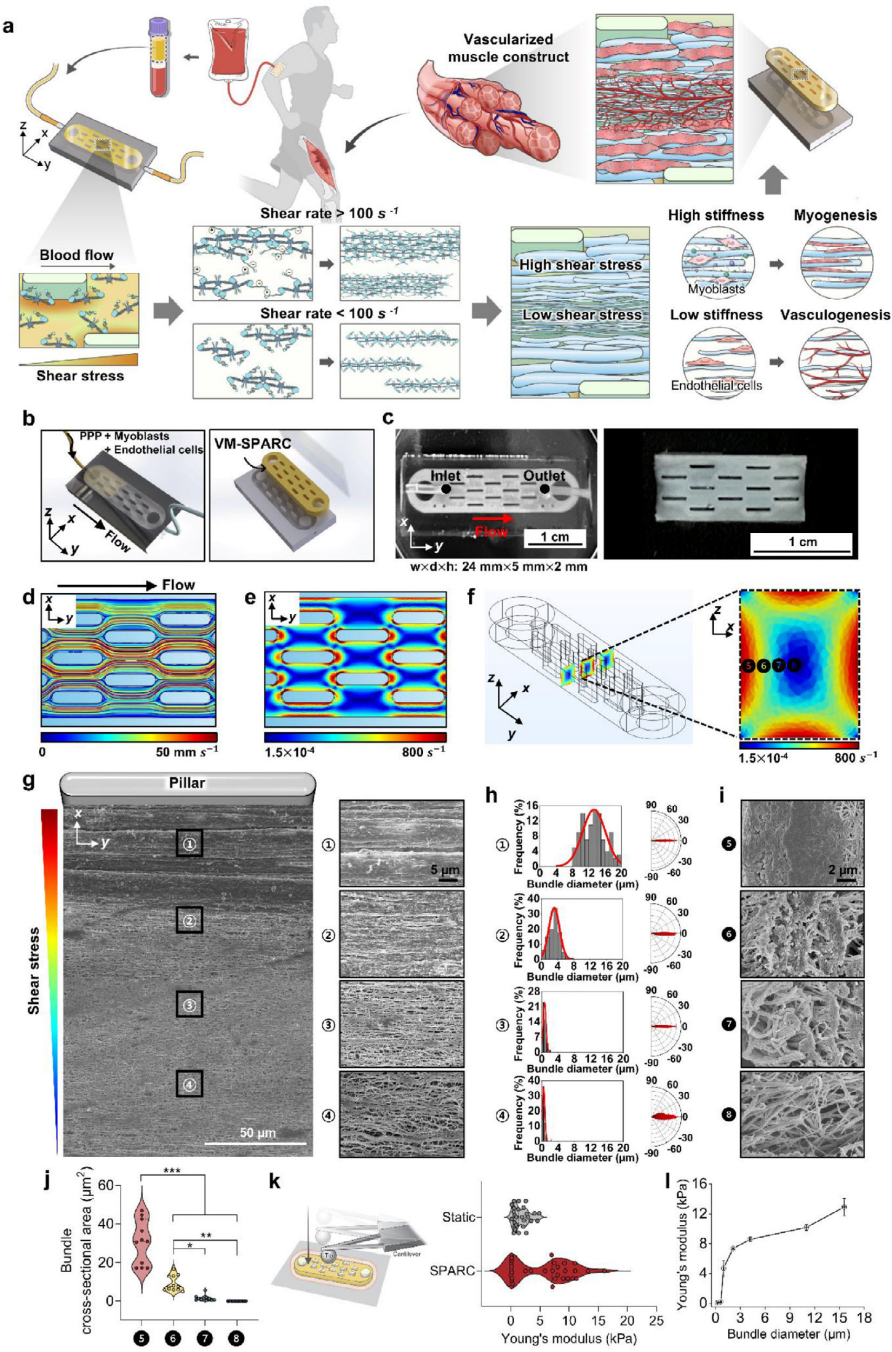
Shear‐proportional fibrin bundling generates an anisotropic and mechanically heterogeneous construct. (a) Schematic of SPARC (Spatio‐chimeric, Plasma‐based, Anisotropic, and shear‐Responsive Construct) formation under spatially defined shear‐stress gradients. Blood plasma flows through a microfluidic channel containing a micropillar array that creates local variations in shear rate, inducing anisotropic fibrin bundling. (b) Schematic of the assembled PDMS microfluidic device with inlet and outlet ports for perfusing platelet‐poor plasma (PPP) containing myoblasts and endothelial cells (left) and schematic of the SPARC after release from the PDMS channel mold (right). (c) Photographs of the fully assembled PDMS device(left) and the demolded SPARC (right) (scale bars = 1 cm). (d) Computational fluid dynamics (CFD) map of streamline simulation of flow through the micropillar geometry, illustrating directionally biased flow trajectories and velocity magnitude (mm s^−1^). (e) Computational simulation of shear‐rate gradients across the pillar array. (f) Cross‐sectional map of shear rate predicted by CFD between adjacent micropillars. (g) Scanning electron microscopy (SEM) images display heterogeneous fibrin‐bundle architecture along the *x*‐axis (scale bar = 50 µm). Right panels: enlarged views of regions 1–4 (scale bar = 5 µm). (h) Fiber diameter distributions across regions 1–4 with polar plots depicting anisotropic fibrin bundle alignment. (i) SEM images of transverse cross‐sections of fibrin bundles in regions 5–8, and (j) corresponding cross‐sectional area (CSA) of fiber bundles by region (*n* = 11). Statistical analysis was performed by one‐way ANOVA followed by post‐hoc Tukey test (*P < 0.05, **P < 0.01, ***P < 0.001 vs. indicated groups). (k) Schematic of the atomic‐force‐microscopy (AFM) setup for local stiffness mapping, and violin plots of Young's modulus for cell‐free SPARC and Static constructs (*n* = 27–32). (l) Correlation between fibrin bundle diameter and local stiffness in cell‐free SPARC. The corresponding local elastic moduli were measured by AFM at matched positions.

To recapitulate the spatial heterogeneity of native muscle, a microfluidic channel with a micropillar array was employed to apply localized shear‐stress gradients during fibrin polymerization, producing SPARC, a structurally anisotropic and mechanically heterogeneous hydrogel (Figure 1a). During the perfusion of platelet‐poor plasma (PPP) containing myoblasts and endothelial cells, the microfluidic device simultaneously enabled in situ fibrin polymerization and cell encapsulation. Following gelation, the construct was demolded from the device as a freestanding hydrogel with sharply defined boundaries, forming a vascularized muscle construct (Figure 1b,c). Computational fluid dynamics (CFD) simulations demonstrated that the micropillar geometry imposed anisotropic streamline patterns within the channel (Figure 1d), generating spatially heterogeneous shear fields with alternating regions of high (>100 s^−1^) and low (<100 s^−1^) shear rates, thereby enabling localized modulation of fibrin assembly dynamics (Figure 1e,f).

The fluidic simulation revealed a gradient of shear rates across the micropillar channel, with locally elevated shear rates (∼800 s^−1^) near the pillar that gradually decreased toward the inter‐pillar region (Figure 1e,f). Scanning electron microscopy (SEM) analysis of cell‐free SPARC confirmed that fibrin bundle morphology changed progressively with local shear rate magnitude, regions subjected to higher shear rates exhibited thicker, densely compacted bundles, whereas those exposed to lower shear rates displayed finer, more loosely assembled fibrils (Figure 1g). Quantitative analysis further validated this graded dependence of bundle diameter on shear rate intensity. Independent of bundle diameter, fibrin fibers aligned consistently parallel to the flow axis, indicating robust shear‐directed organization (Figure 1h). As the flow rate increased, the resulting higher shear stress further enhanced fibrin fiber alignment within the device (Figure S1). Moreover, because the local fluid shear rate decreases toward the channel centerline, the fibrin bundle thicknesses were predicted to scale with the local shear rate across the channel cross‐section (Figure 1f,i). SEM images of transverse fibrin‐bundle cross‐sections, together with quantitative analyses, corroborated the predicted three‐dimensional, shear‐rate‐dependent morphology–thicker bundles at the higher‐shear periphery and thinner fibers in the lower‐shear core (Figure 1i,j).

We next explored the relationship between fibrin bundle architecture and local elastic modulus in cell‐free SPARC by atomic force microscopy (AFM) nanoindentation, confirming that shear‐dependent fibrin organization generates localized mechanical heterogeneity (Figure 1k,l). SPARC displayed a broad Young's modulus distribution, with stiffer, bundle‐rich high‐shear regions in the kilopascal range (2.5–16.06 kPa) and softer lower‐shear domains in the pascal range (15.85–300.16 Pa). In contrast, Static constructs–formed under identical geometric confinement but without flow‐induced shear–exhibited a narrow modulus distribution centered at the lower range. The mechanical heterogeneity of SPARC offers a spatio‐chimeric, dual‐stiffness microenvironment—≈10–250 Pa and ≈10–50 kPa—consistent with ranges reported to promote endothelial morphogenesis and myogenic differentiation, respectively [5, 13, 14]. This supports that the shear‐modulated fibrin network simultaneously spans the mechanical spectrum conducive to both vascular and myogenic maturation. SPARC, in turn, encodes shear‐proportional fibrin bundling, generating multiscale structural and mechanical heterogeneity that guides spatially organized cell maturation. Mechanistically, shear flow promotes kinetic alignment of fibrin fibers, increasing the frequency of parallel encounters and facilitating lateral association [1, 15, 16]. Our theoretical model further corroborates shear‐induced fibrin bundling and stiffness modulation by quantifying diffusion‐ versus shear‐driven collision rates across relevant length scales. (Figure S2). Simultaneously, mechanical extension of fibrin strands exposes cationic residues—lysine (Lys^+^) and arginine (Arg^+^)—within the flexible αC domains [17, 18, 19], enhancing electrostatic interactions that stabilize lateral fiber association [20, 21]. Increasing the ionic strength of PPP by adding ≥50 mm NaCl attenuated electrostatic interactions, and consequently, reduced bundle formation, yielding thinner, more disordered fibrils under the same flow‐induced polymerization conditions (Figure S3). This response reflects a charge‐screening effect, whereby chloride ions neutralize exposed Lys^+^/Arg^+^ sites and suppress αC–αC electrostatic bridging [22]. These findings are consistent with previous SAXS and molecular dynamics studies reporting strain‐dependent extension and charge redistribution within fibrin αC regions [23, 24]. Collectively, these results suggest that shear‐induced fibrin bundling arises from a coupled hydrodynamic–electrostatic mechanism, in which hydrodynamic alignment organizes protofibrils and electrostatic interactions stabilize lateral assembly under shear.

### 2.2 Shear‐Directed Anisotropy Promotes Cellular Alignment and Myogenic Progression

**FIGURE 2:**
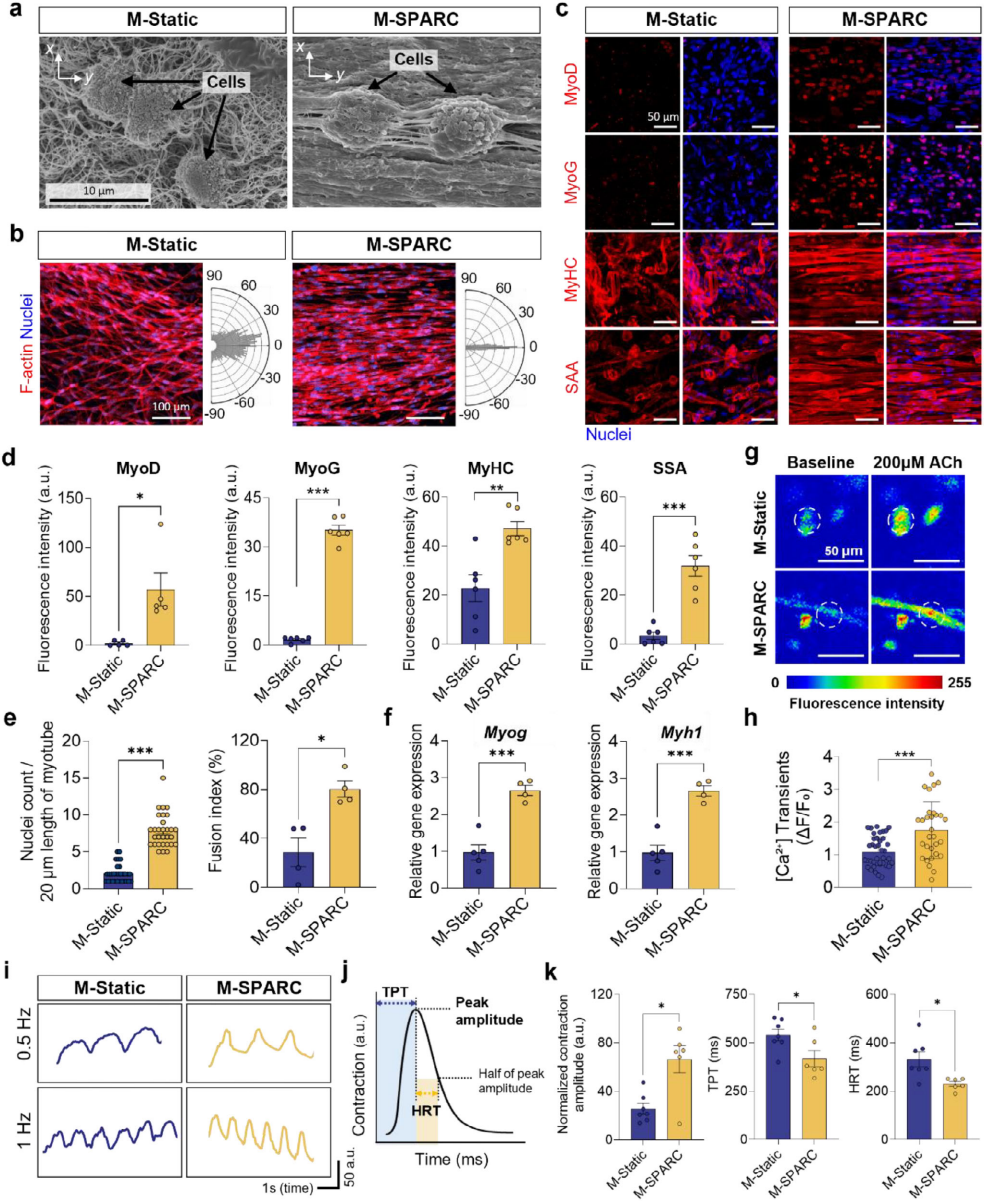
Mechanical and structural guidance of myogenic differentiation in SPARC. (a) SEM images immediately after cell seeding (day 0) of randomly oriented myoblasts and fibrin networks in 3D fibrin constructs formed in a static condition (M‐Static) and shear‐assembled muscle constructs (M‐SPARC) (scale bar = 10 µm). (b) Fluorescence images of filamentous actin (phalloidin, red) and nuclei (DAPI, blue) at day 3 (scale bars = 100 µm). Angular distribution plots compare cytoskeletal orientation for the cells within M‐Static and M‐SPARC. (c) Immunostaining for myogenic markers, MyoD, MyoG, MyHC, and SAA, at 6 days of culture (scale bars = 50 µm). (d) Quantification of fluorescence intensity for all four myogenic markers in M‐Static and M‐SPARC (*n* = 5–6). (e) Quantification of myonuclei per myotube (*n* = 30) and fusion index (*n* = 4). (f) qRT‐PCR analysis of *Myog* and *Myh1* expression in M‐Static and M‐SPARC (*n* = 4). (g) Fluo‐4 imaging of acetylcholine (200 µm)‐induced [Ca^2^^+^] responses. Δ*F*/*F*_0_ fluorescence signals are shown for defined regions of interest (scale bars = 50 µm). (h) Fluo‐4 calcium imaging of acetylcholine (200 µm)‐induced responses, showing [Ca^2^^+^] transients (Δ*F*/*F*_0_) quantified from defined regions of interest (*n* = 30–40). (i) Representative contraction traces under field stimulation at 0.5 and 1 Hz (5 V). (j) Schematic of twitch contraction kinetics showing time to peak tension (TPT) and half‐relaxation time (HRT). (k) Quantification of normalized contraction amplitude, TPT, and HRT (*n* = 6). All statistical analyses were determined using unpaired *t*‐tests (*P < 0.05, **P < 0.01, ***P <0.001 vs. M‐Static group).

The effect of shear‐directed matrix anisotropy on myoblast organization and maturation was examined using fibrin constructs containing only myogenic cells, generated either under static conditions (M‐Static) or under shear flow using the SPARC system (M‐SPARC). In M‐SPARC, SEM imaging immediately after cell seeding (day 0) revealed myoblasts oriented parallel to the fibrillar axis, whereas cells in the M‐Static construct, patterned by lateral pillars under passive tension, exhibited random orientation (Figure 2a, Figure S4). By day 3, immunofluorescence staining for filamentous actin (F‐actin) revealed pronounced alignment along the anisotropic fibrin bundles in M‐SPARC (Figure 2b). Angular distribution analysis quantitatively confirmed coordinated anisotropy in M‐SPARC compared with the disorganized orientation observed in M‐Static constructs.

Myogenic progression was analyzed in high‐shear regions of M‐SPARC and matched peripheral zones of M‐Static constructs by immunostaining for myogenic markers, including myogenic differentiation 1 (MyoD1), myogenin (MyoG), myosin heavy chain (MyHC), and sarcomeric α‐actinin (SAA). Myoblasts in M‐SPARC showed enhanced expression of all markers with well‐defined sarcomeric organization relative to those in the M‐Static construct (Figure 2c,d). Quantitative fluorescence analysis verified significantly increased expression of these markers, accompanied by elevated myonuclear content and fusion index (Figure 2e). The higher number of nuclei per myotube and increased proportion of multinucleated fibers indicate more efficient fusion and structural maturation within M‐SPARC. Consistently, qRT‐PCR confirmed transcriptional upregulation of *Myog* and *Myh1* by 2.05 ± 0.13‐ and 3.04 ± 0.71‐fold, respectively, relative to M‐Static, demonstrating progression toward late‐stage myogenic differentiation (Figure 2f).

Functional maturation was evaluated by monitoring calcium transients using Fluo‐4, a fluorescent Ca^2^^+^ indicator whose fluorescence increases upon calcium binding. Upon acetylcholine (ACh) stimulation (200 µm), M‐SPARC exhibited calcium waves with significantly higher peak fluorescence intensity compared to static controls, indicating more efficient Ca^2^^+^ handling during excitation–contraction coupling (Figure 2g,h). Quantification of fluorescence changes (Δ*F*/*F*_0_) from defined regions of interest confirmed significantly enhanced calcium transient amplitudes in M‐SPARC.

Consistently, M‐SPARC exhibited stronger contractile responses under electric field stimulation compared with M‐Static constructs. Representative contraction traces recorded at 0.5 and 1 Hz (5 V) demonstrated larger and more regular twitch responses in M‐SPARC (Figure 2i). Quantitative analysis revealed significantly higher normalized contraction amplitudes together with faster twitch kinetics, reflected by shortened time to peak tension (TPT), defined as the time required to reach maximal contraction, and reduced half‐relaxation time (HRT), which represents the time required for the contraction amplitude to decay to 50% of the peak value (Figure 2j,k). These parameters collectively indicate improved excitation–contraction coupling and enhanced functional maturation of the engineered myotubes.

### 2.3 Spatial Divergence of Myogenic and Endothelial Lineages within Anisotropic, Mechanically Bimodal SPARC

**FIGURE 3:**
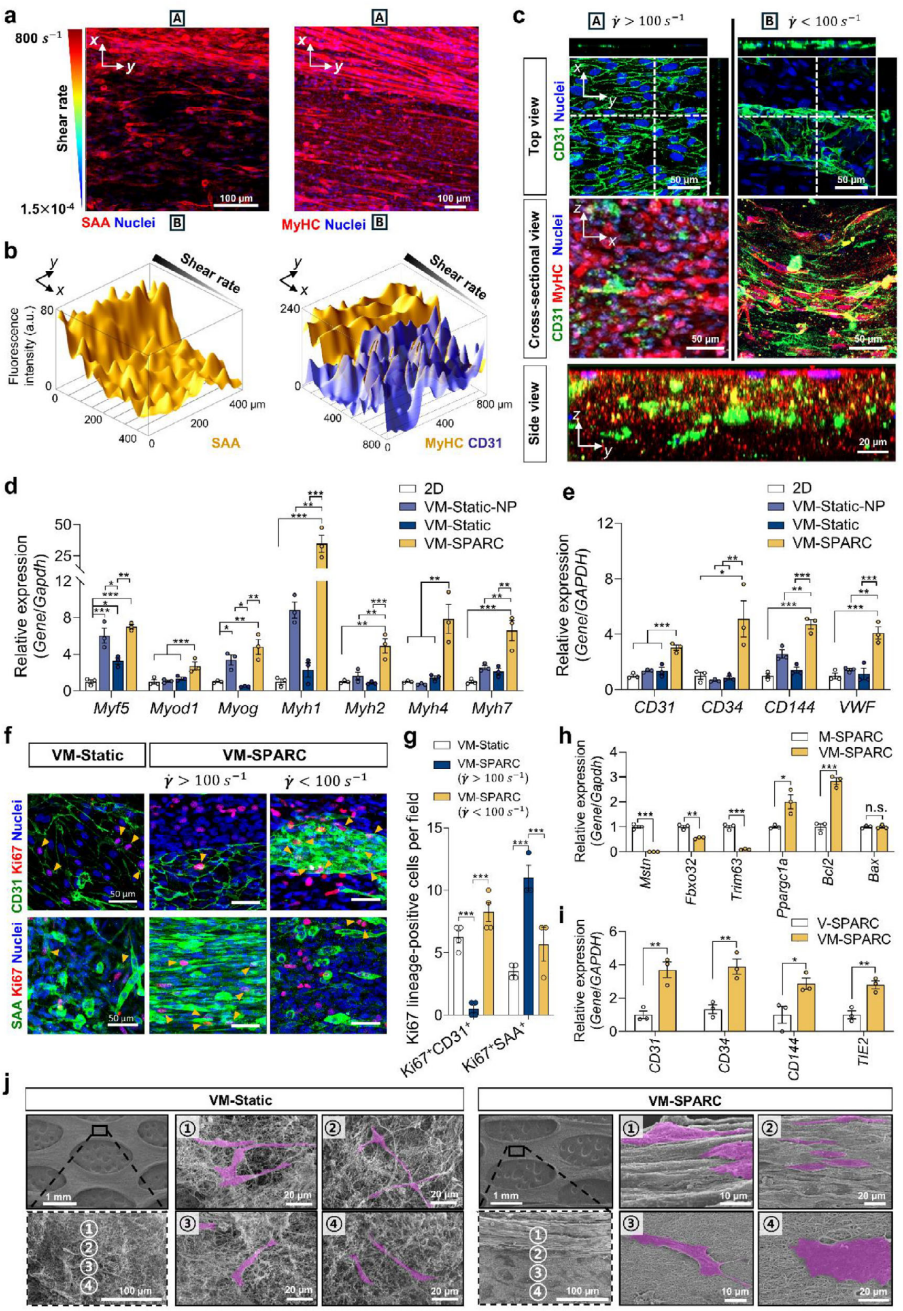
Spatial divergence in myogenic and endothelial responses within SPARC. (a) Representative immunofluorescence images of SAA (left) and MyHC (right) with nuclei counterstained in blue (scale bars = 100 µm). A = high‐shear region near a micropillar; B = lower‐shear region between micropillars. (b) Quantitative analysis of SAA (left), MyHC, and CD31 (right) fluorescence intensity from region A to B, demonstrating reciprocal signal distribution along the shear axis. (c) Immunofluorescence images of MyHC (red), CD31 (green), and nuclei (blue) in higher‐ and lower‐shear regions, presented as a top‐view (*x‐*‐*y* plane) and orthogonal projections–*z*–*x* (middle) and *z‐*‐*y* (bottom) (scale bars = 150, and 20 µm). (d) qRT‐PCR analysis of myogenic regulators (*Myod1*, *Myf5*, *Myog*) and late structural markers (*Myh1/2/4/7*) in VM‐SPARC compared with control groups (2D culture, VM‐Static‐NP, and VM‐Static) (*n* = 3). (e) qRT‐PCR analysis of vascular markers (*CD31*, *CD34*, *CD144*, and *VWF*) in VM‐SPARC and control constructs (*n* = 3). (f) Ki67 co‐staining with lineage markers in VM‐Static and VM‐SPARC. Endothelial cells were identified by CD31 and myogenic cells by SAA (scale bars = 50 µm). Yellow arrowheads indicate Ki67^+^ cells co‐localized with lineage markers. (g) Quantification of Ki67^+^CD31^+^ endothelial cells and Ki67^+^SAA^+^ myogenic cells across conditions (*n* = 4). (h) qRT‐PCR analysis of myogenic gene expression in myogenic‐only constructs (M‐SPARC) and co‐cultured constructs (VM‐SPARC) (*n* = 3). (i) qRT‐PCR analysis of endothelial markers in endothelial‐only constructs (V‐SPARC) and VM‐SPARC (*n* = 3). (j) SEM images of VM‐Static (left) and VM‐SPARC (right) constructs after 7 days of culture. Low‐magnification images show overall construct morphology (scale bars = 1 mm, 100 µm), and numbered regions correspond to higher‐magnification views of cells (magenta) within the fibrin microstructure (scale bars = 10–20 µm). Statistical analyses were performed using one‐way ANOVA with Tukey's post hoc test (d,e,g) or unpaired *t*‐test (h,i). (*p < 0.05, **p < 0.01, ***p < 0.001).

The anisotropic and mechanically bimodal architecture of SPARC established distinct yet complementary microenvironments that supported the concurrent maturation of myogenic and endothelial populations within co‐culture, shear‐assembled constructs that exhibit vascularized muscle‐like organization (VM‐SPARC). Immunofluorescence staining for SAA and MyHC revealed a gradient of myogenic differentiation along the shear‐rate gradient axis (Figure 3a). In high‐shear regions (region A, >100 s^−^^1^), SAA^+^ and MyHC^+^ myotubes exhibited pronounced alignment along the fibrillar axis, whereas weaker signals were observed in the lower‐shear regions (region B, <100 s^−^^1^). Quantitative fluorescence analysis further confirmed this reciprocal organization, showing higher SAA and MyHC intensities in high‐shear regions and elevated CD31 signals in low‐shear regions (Figure 3b).

By contrast, in compliant, low‐shear regions, endothelial cells organized into branched, network‐like structures characteristic of early vascular morphogenesis, whereas in higher‐shear domains, they formed aligned monolayers interwoven with myotubes (Figure 3c). Orthogonal side‐view (*y–z* plane) imaging further demonstrated vertical stratification: myogenic cells occupied the stiffer, thicker bundle‐rich zones, while endothelial cells populated softer layers conducive to lumen formation.

Gene expression profiling was performed to evaluate lineage specification of myoblasts and endothelial cells cultured in 2D monolayers and in 3D co‐culture constructs, including static constructs with no pillar structures (VM‐Static‐NP), pillar‐patterned static constructs (VM‐Static), and VM‐SPARC. Notably, VM‐Static‐NP constructs underwent pronounced contraction during culture, leading to a substantial reduction in construct size and loss of defined scaffold geometry (Figure S5). Among the tested conditions, VM‐SPARC exhibited the strongest lineage specification under shear‐aligned environments (Figure 3d,e). VM‐SPARC exhibited the highest expression of early myogenic regulators (*Myf5*, *Myod1*), the differentiation marker (*Myog*), and late‐stage structural isoforms (*Myh1*, *Myh2*, *Myh4*, *Myh7*), indicating advanced myotube maturation (Figure 3d). Notably, *Myh1*, a fast‐twitch isoform, was upregulated by more than 30‐fold relative to 2D culture. Endothelial‐associated genes, including *PCAM1* (*CD31*), *CD34*, *VE‐Cadherin* (*CD144*), and *von Willebrand factor* (*VWF*), were also markedly elevated (Figure 3e) in VM‐SPARC. CD31 and CD144 are adhesion molecules essential for endothelial junction integrity [25], CD34 marks vascular progenitor cells [26], and VWF represents a canonical endothelial functional marker [27]. The coordinated upregulation of these endothelial markers indicates augmented endothelial differentiation and morphogenesis in VM‐SPARC, reflecting the establishment of permissive microdomains that promote vascular lineage maturation. Consistent with these findings, immunostaining revealed spatially organized endothelial structures within the constructs. CD31^+^ and CD34^+^ endothelial networks were predominantly observed in the low‐shear regions of VM‐SPARC, whereas VM‐Static exhibited minimal CD31^+^ or CD34^+^ organization (Figure S6).

The proliferative behavior of each cell population was next assessed at day 3 of culture by Ki67 co‐staining with lineage‐specific markers (Figure 3f; Figure S7). Quantification of CD31^+^Ki67^+^ endothelial cells and SAA^+^Ki67^+^ myogenic cells revealed region‐dependent differences in VM‐SPARC (Figure 3g). Individual fluorescence channels are shown separately to clarify marker localization (Figure S7). The compliant low‐shear regions contained a greater number of CD31^+^Ki67^+^ endothelial cells, whereas the stiffer high‐shear regions exhibited a higher abundance of SAA^+^Ki67^+^ myogenic cells. In contrast, VM‐Static constructs showed comparable numbers of proliferative endothelial and myogenic cells, similar to those observed in the low‐shear regions of VM‐SPARC. These findings suggest that the spatially programmed microenvironment of SPARC is associated with region‐specific growth patterns of endothelial and myogenic cells during early culture.

Transcriptional analysis performed at day 7 further revealed interactions between the two cell populations. Gene expression profiles were compared among shear‐assembled vasculature‐like constructs (V‐SPARC), M‐SPARC, and VM‐SPARC. Myogenic gene expression analysis revealed coordinated changes associated with improved myogenic cell maintenance under co‐culture conditions (Figure 3h). Muscle atrophy‐associated genes (*Mstn*, *Trim63*, and *Fbxo32*) were reduced in VM‐SPARC compared with M‐SPARC, whereas expression of the mitochondrial oxidative metabolism regulator *Ppargc1a* and the cell survival‐associated gene *Bcl2* was increased. In contrast, the pro‐apoptotic gene *Bax* showed no significant difference relative to mono‐culture conditions. These findings suggest reciprocal interactions between endothelial and myogenic cells that support metabolic activity and survival within the SPARC microenvironment. Conversely, endothelial lineage markers, including *CD31*, *CD34*, *CD144*, and *TIE2* were significantly increased in VM‐SPARC compared with V‐SPARC, suggesting that the presence of myogenic cells promotes endothelial maturation or stabilization within the co‐culture environment (Figure 3i).

Live/Dead staining confirmed consistently high cell viability (>90%) across M‐SPARC, V‐SPARC, and VM‐SPARC groups, indicating that neither co‐culture conditions nor shear‐derived mechanical environments adversely affected cell survival (Figure S8). After extended in vitro culture for 7 and 14 days, SEM analysis confirmed that the aligned fibrin architecture of VM‐SPARC remained structurally preserved, with no evidence of collapse or loss of anisotropy (Figure 3j; Figure S9). Notably, the spatial differences in fiber organization between high‐ and low‐shear regions were maintained over time, indicating that the shear‐programmed microstructure and associated stiffness gradients are stable during prolonged culture. Together, these findings demonstrate that the anisotropic mechanical bimodality within VM‐SPARC provides dual lineage‐specific cues, promoting myogenic differentiation in stiff, high‐shear domains while simultaneously facilitating endothelial morphogenesis in compliant, low‐shear regions, thereby reproducing the spatial organization characteristic of native vascularized muscle.

### 2.4 VM‐SPARC Promote Muscle Regeneration and Functional Recovery in a Mouse VML Model

SPARC scaffolds exhibited biocompatibility suitable for implantation. Scaffold degradability and the cytocompatibility of its degradation products were evaluated (Figure S10). Cell‐free SPARC incubated in PBS, serum‐containing medium (10% FBS), or plasmin solution (0.1 U mL^−^^1^) at 37°C showed progressive degradation, with the most rapid mass loss observed under fibrinolytic conditions. After 7 days, relative scaffold weight decreased to 11.2%, 13.3%, and 7.2% in PBS, serum‐containing medium, and plasmin solution, respectively (Figure S10a,b). Cytocompatibility of scaffold degradation products was assessed using conditioned media collected from PBS and serum‐containing conditions, and no detectable cytotoxicity was observed in the myogenic/endothelial cell co‐culture model (Figure S10c).

In vivo biocompatibility was evaluated by subcutaneous implantation of cell‐free SPARC scaffolds followed by analysis after one week (Figure S11). Hematoxylin and eosin (H&E) staining showed host cell infiltration into the scaffold region while preserving overall tissue architecture without necrosis (Figure S11a). Masson's trichrome (MT) staining revealed no excessive collagen deposition or fibrotic encapsulation compared with the Sham group (Figure S11a,b). Toluidine blue (TB) staining showed a modest increase in mast cells at this early time point (Figure S11a,c), whereas Ly6G^+^ neutrophil levels were comparable between Sham and SPARC groups (Figure S11d,e). CD86^+^/CD68^+^ macrophages were detected within the implantation region (Figure S11f,g), and the M1 polarization ratio remained comparable between the Sham and SPARC groups, indicating no evident pro‐inflammatory macrophage activation. Serum AST and ALT levels remained within normal physiological ranges, indicating the absence of systemic toxicity (Figure S11h).

**FIGURE 4:**
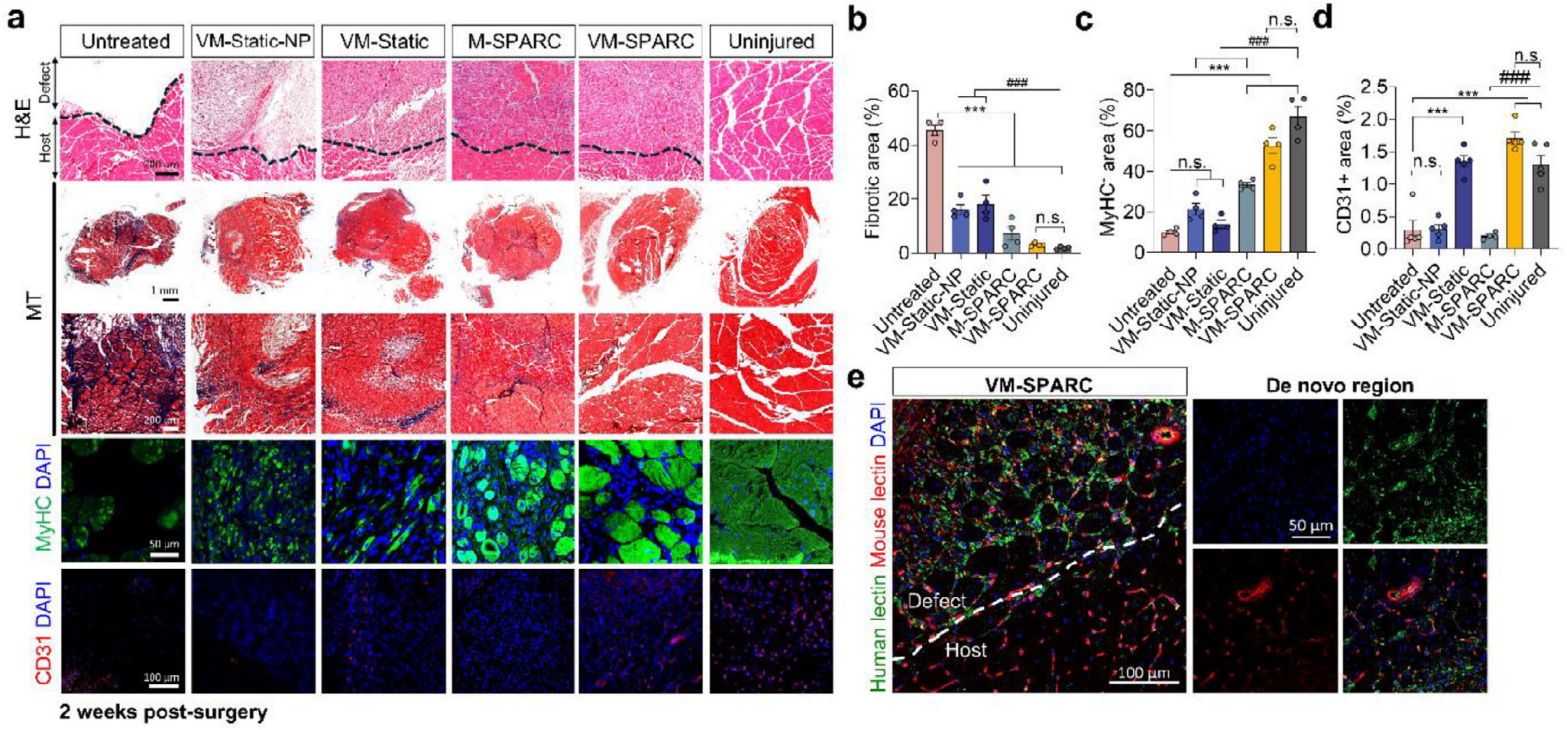
Accelerated muscle regeneration at 2 weeks post‐transplantation of VM‐SPARC. (a) Representative histology (H&E, MT) and immunohistostaining (MyHC, CD31) of defect regions from Untreated, VM‐Static‐NP, VM‐Static, M‐SPARC, VM‐SPARC, and Uninjured groups (scale bars: H&E = 200 µm; MT = 1 mm and 200 µm; MyHC = 50 µm; CD31 = 100 µm). (b–d) Quantification of fibrotic area (*n* = 4) (b), MyHC^+^ area (*n *= 4) (c), and CD31^+^ area (*n* = 5) (d) across groups measured from immunostained sections. All statistical analyses were determined using one‐way ANOVA followed by post‐hoc Tukey test (***p < 0.001 vs. Untreated, and ###*P *< 0.001 vs. Uninjured groups). (e) Immunofluorescence image of vascular anastomosis using human‐specific lectin (green), mouse‐specific lectin (red), and DAPI (blue). Co‐localized signal indicates connection between human and host vasculature (scale bar = 100 µm; inset scale bars = 50 µm).

Implantation of VM‐SPARC into a mouse VML model accelerated regenerative remodeling and vascular integration. At two weeks post‐implantation, histological analyses by H&E and MT staining revealed early muscle repair and markedly reduced fibrosis in the VM‐SPARC compared with VM‐Static‐NP, VM‐Static, and untreated controls (Figure 4a,b). Immunohistochemical staining showed a significantly larger MyHC^+^ area, with nascent myofiber clusters exhibiting bundle‐like organization along the defect axis, indicative of early myogenic assembly despite their immature morphology (Figure 4a,c). In contrast, VM‐Static‐NP, and VM‐Static grafts displayed scattered, disorganized MyHC^+^ cells, while untreated defects showed minimal MyHC^+^ signal. CD31^+^ endothelial density was also substantially higher in VM‐SPARC (Figure 4a,d). Dual fluorescent lectin labeling using human‐ and mouse‐specific probes demonstrated co‐localized staining within the defect region, confirming vascular anastomosis between implanted human endothelial cells in VM‐SPARC and host murine vessels. Because lectins bind only luminal glycans accessible from the circulation, this colocalization verified perfusion, indicating early functional coupling of VM‐SPARC with the host vasculature (Figure 4e). Notably, M‐SPARC, which was cultured in SPARC but contained only myogenic cells without endothelial cells, also showed reduced fibrosis and increased MyHC^+^ area compared with other control groups at two weeks post‐implantation. However, MyHC expression and vascular density remained significantly lower than those observed in VM‐SPARC. These results suggest that while the SPARC microenvironment supports early muscle repair, rapid vascular formation plays a critical role in promoting efficient muscle regeneration during the early healing phase.

**FIGURE 5:**
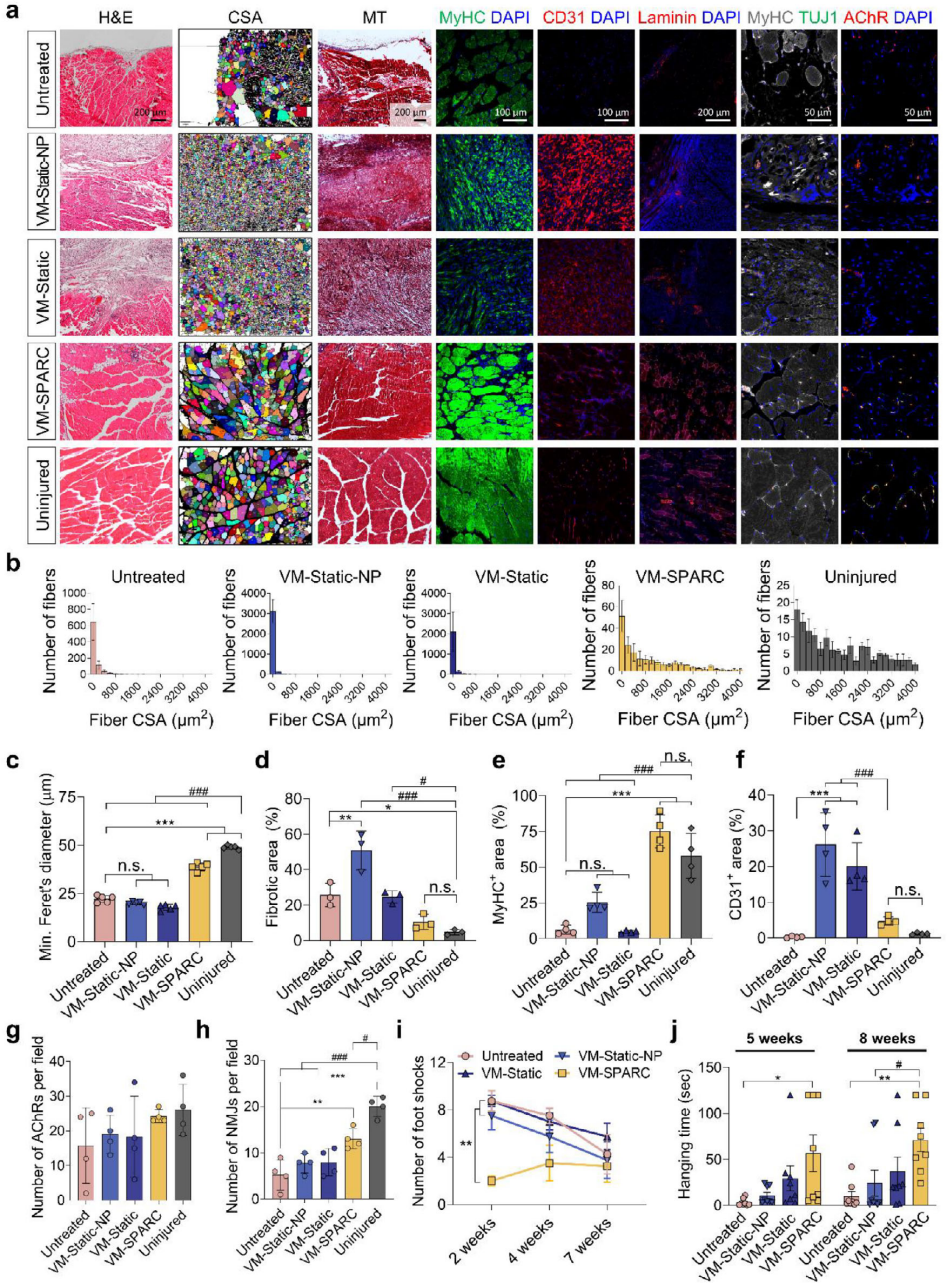
VM‐SPARC implantation promotes sustained muscle regeneration and functional recovery 8 weeks post‐VML injury. (a) Representative histology (H&E, MT) and immunohistochemical staining (MyHC, CD31, laminin, TUJ1, AChR) of regenerated muscle tissue within the defect region (scale bars = 50, 100, and 200 µm). (b) Frequency distribution of myofiber CSA across groups. (c–f) Quantification of minimum Feret's diameter (*n* = 5) (c), fibrotic area (*n* = 3) (d), MyHC^+^ area (*n* = 3) (e), and CD31^+^ area (*n* = 4) (f) for all groups. (g,h) Quantitative analysis shows the number of AChR‐positive sites (*n* = 4) (g) and the number of NMJ sites (*n* = 4) (h) across experimental groups. NMJ sites were defined as regions where AChR clusters overlapped with neuronal marker (TUJ1) and MyHC signals. (i,j) Functional assessment by treadmill fatigue test (*n* = 4) (i) and horizontal bar hanging test (*n* = 8) (j) in VM‐SPARC, VM‐Static, VM‐Static‐NP, and Untreated groups. Statistical analyses were performed using one‐way ANOVA followed by post hoc Tukey's test (c–i) or two‐way ANOVA followed by post hoc Tukey's test (j). (*p < 0.05, **p < 0.01, ***p < 0.001 versus Untreated, and #*p* < 0.05, ###*p* < 0.001 versus Uninjured groups).

By 8 weeks, VM‐SPARC supported extensive tissue remodeling characterized by mature muscle architecture and restored vascular organization. H&E staining revealed densely organized myofiber bundles with continuous tissue integration across the defect, whereas VM‐Static‐NP, VM‐Static, and untreated groups retained disorganized architecture (Figure 5a). Cross‐sectional morphometry demonstrated a clear shift toward larger myofiber CSA in VM‐SPARC‐treated defects, closely resembling uninjured muscle (Figure 5a,b), and measurement of minimum Feret's diameter confirmed advanced structural maturation compared with all controls (Figure 5c). MT staining showed minimal interstitial collagen deposition in VM‐SPARC, in contrast to pronounced fibrosis in VM‐Static‐NP, VM‐Static, and untreated groups. Quantitative analysis confirmed the fibrotic area in VM‐SPARC was reduced to near‐physiological levels, showing a significant decrease relative to VM‐Static‐NP and VM‐Static constructs (Figure 5a,d).

Immunohistochemistry further revealed densely organized MyHC^+^ fibers resembling native morphology, with the MyHC^+^ area significantly higher in VM‐SPARC than in all control groups and approaching uninjured values (Figure 5a,e). The CD31^+^ area was markedly elevated in VM‐Static‐NP and VM‐Static constructs at eight weeks, indicating persistent and disorganized endothelial accumulation rather than functional vascular remodeling, whereas VM‐SPARC exhibited CD31^+^ distribution patterns suggestive of more organized vascular structures comparable to uninjured muscle (Figure 5a,f). Laminin staining confirmed restoration of continuous basal lamina outlining regenerated myofibers in VM‐SPARC, contrasting with the fragmented or absent laminin observed in the control groups (Figure 5a). Neuromuscular junction (NMJ)‐related structures were also observed within the regenerated tissue (Figure 5a). AChR clusters were detected across all groups, and the number of AChR‐positive sites was comparable among conditions, with slightly higher values in VM‐SPARC and Uninjured muscle, although not significant (Figure 5g). NMJ density was quantified by counting sites showing spatial colocalization of AChR, neuronal marker, and MyHC signals. Only the VM‐SPARC group exhibited significantly higher NMJ density than the Untreated group, whereas other treated groups showed no significant difference (Figure 5h). NMJ density in VM‐SPARC remained lower than that of uninjured muscle, indicating partial neuromuscular reconstruction within the regenerated tissue.

Functional recovery was first evaluated using a treadmill endurance test, a widely used assay to assess locomotor capacity and fatigue resistance in rodent muscle injury models [28]. Because the injury was induced in the hindlimb, treadmill performance reflects the ability of the affected musculature to sustain propulsion and weight‐bearing during repetitive loading. VM‐SPARC‐treated mice exhibited significantly reduced foot‐shock counts compared to control groups (Figure 5i), indicating improved endurance capacity and functional recovery of the injured hindlimb muscles. We next performed a horizontal‐bar hanging test to evaluate muscle force generation and suspension endurance [29]. This assay requires sufficient muscle force and coordinated limb engagement to support body weight for a sustained period and is commonly used to assess in vivo muscle performance. VM‐SPARC‐treated mice demonstrated significantly prolonged hanging times (Figure 5j), further supporting functional restoration following VML repair. Collectively, these findings demonstrate that VM‐SPARC promotes long‐term structural remodeling, vascular restoration, and functional recovery following volumetric muscle loss.

## 3 Conclusion

Functional muscle regeneration requires simultaneous reconstruction of the structural, mechanical, and vascular complexity inherent to native tissue—an integration that remains a major challenge in current biofabrication strategies. The limited regenerative capacity of large muscle grafts is primarily due to the absence of perfusable architecture and mechanically supportive environments, leading to rapid cell loss and impaired maturation [30]. While several previously reported muscle graft designs incorporate structural alignment or biomechanical support [31, 32, 33], they have not captured the dual mechanical and architectural integration necessary for coordinated myogenic and vascular regeneration.

The SPARC system addresses these limitations by creating spatially encoded, bimodal mechanical properties within an anisotropic microenvironment. By harnessing shear‐driven fibrin bundling, which dominates over diffusion‐mediated polymerization at micrometer scales SPARC establishes controlled anisotropy and spatially bimodal mechanical properties: stiff, anisotropic regions conducive to myogenic differentiation and compliant, low‐shear regions permissive to endothelial morphogenesis. This multiscale organization recapitulates the spatial hierarchy of native muscle, guiding compartmentalized cell fate decisions without synthetic modification or external mechanical modulation.

When applied in vivo, VM‐SPARC promoted rapid integration, minimized fibrosis, and enabled long‐term restoration of muscle architecture, vascular perfusion, and functional performance in a rodent VML model. Collectively, these findings position SPARC as a clinically relevant, multiscale biofabrication strategy for reconstructing vascularized muscle tissue. By coupling hydrodynamic alignment with endogenous plasma biochemistry, this platform establishes a new framework for functionally and mechanically guided tissue regeneration and holds promise for future applications in engineered tissues that require spatially coordinated, multimodal mechanical properties. Future incorporation of patient‐derived endothelial cells or endothelial progenitor cells, together with iPSC‐derived myogenic progenitors, may further improve physiological relevance and enhance the translational potential of this platform.

## 4 Experimental Section

### 4.1 Platelet‐Poor Plasma Preparation

All animal and human blood collection protocols were approved by the Institutional Animal Care and Use Committee (IACUC) and the Institutional Review Board (IRB) of Ulsan National Institute of Science and Technology (UNIST). Following IRB approval (UNISTIRB‐19‐23‐C), human platelet‐poor plasma was prepared by thawing fresh frozen plasma (FFP) obtained from the Korea Red Cross.

### 4.2 Device Fabrication

Computer numerical control (CNC) machining was used to fabricate the poly(methyl methacrylate) (PMMA) mold for replicating polydimethylsiloxane (PDMS) channel structures. A PDMS prepolymer was mixed with a curing agent at a ratio of 10:1 and poured onto a PMMA mold. After curing at 80°C for 2 h, the micropatterned PDMS was peeled from the PMMA mold. Inlet and outlet ports were made at the side of the PDMS slabs to allow liquid to flow through the channel. And then, the PDMS slab without the micropillar array spot was coated with the uncured PDMS prepolymer and temporarily attached to the glass at a 180°C hotplate for 1 min. The PDMS channel was treated with ethanol to sterilize and prevent air bubbles from being trapped in the device. Subsequently, the ethanol was washed out with deionized (DI) water, and a 0.5% Pluronic solution was used to easily detach the SPARC from the PDMS channel.

### 4.3 Computational Fluid Dynamics Simulation

To predict the blood plasma flows and analyze shear rates, COMSOL Multiphysics software (COMSOL Inc., MA, USA) was used. The simulation includes the entire microfluidic channel geometry described in Figure 1. The blood was assumed to be a 2D, incompressible, and non‐Newtonian fluid. To characterize the non‐Newtonian behavior of blood plasma, a generalized power–law constitutive equation for viscosity was used, with parameters based on published values. The flow rate (860 mL h^−1^) as an inlet boundary condition and constant atmospheric pressure at the outlet boundary were defined for the model.

### 4.4 SPARC Formation

The 1 mL human plasma was recalcified with 5 mm CaCl_2_ before flowing through the PDMS device. A peristaltic pump (Ismatec Reglo ICC Independent‐Channel Control Peristaltic Pump, ISMATEC, Wertheim, Germany) controlled the blood flow rate perfusing through the channel. The flow rate gradually reduced from 840 mL h^−1^ as the engineered SPARC was progressively developed for 15 min. After finishing the SPARC formation in the microfluidic device, the glass slide was removed from the PDMS substrate, leaving the SPARC exposed to air. To construct the Static and Static‐NP constructs, 5 mm CaCl_2_‐added plasma was immediately introduced into the PDMS device with or without micropillar arrays, respectively.

### 4.5 Scanning Electron Microscope

M‐SPARC were imaged immediately after fabrication (day 0), whereas VM‐SPARC were analyzed after 7 and 14 days of culture. The SPARC obtained from the microfluidic device was rinsed with a saline solution and fixed with a 2.5% glutaraldehyde solution for 1 h. After washing with DI water three times, the sample was dehydrated following serial treatments with ethanol solutions of 25%, 50%, 75%, 95%, and 100%. Subsequently, a mixture of hexamethyldisilane (HMDS) and ethanol (1:1) was used for dehydration, and then the 100% HMDS treatment completely dried the samples, which were stored in a vacuum desiccator until SEM imaging. Before imaging, a thin gold–palladium film was deposited on the sample pieces (20 mA for 60 s) with a Hitachi sputter coater (MC1000 Ion Sputter Coater, Hitachi High‐Technologies Corporation, Tokyo, Japan). Images were taken by the Cold FE‐SEM (Model No. S‐4800, Hitachi High‐Technologies Corporation) at an accelerating voltage of 7 kV. The average diameter and direction of fibers were analyzed by the ImageJ software (NIH, USA).

### 4.6 AFM Analysis of SPARC Dynamic Stiffness

We characterize the hydrogel stiffness by the Young's modulus using atomic force microscopy (AFM). The experiments were carried out using a commercial AFM (NX10, Park Systems, Korea) equipped with a 1 µm diameter silicon bead AFM probe (Novascan Technologies, IA, USA). The cantilever spring constant and deflection sensitivity were calibrated in fluid via the thermal noise method. Force measurements were then carried out in five random regions of interest (ROI) in one SPARC structure. The elastic modulus for each force curve was calculated using the XEI program (Park Systems).

### 4.7 Cell Culture

C2C12 (ATCC, VA, USA) were grown in the high‐glucose Dulbecco's Modified Eagle Medium (DMEM) supplemented with 10% Fetal Bovine Serum (FBS) and 1% Penicillin‐Streptomycin (Pen‐Strep). Human umbilical vein endothelial cells (Sartorius, Göttingen, Germany) were grown in endothelial cell medium (ScienCell, CA, USA). For fibroblast‐conditioned medium, normal human lung fibroblasts (Lonza, Basel, Switzerland) were cultured in DMEM with 10% FBS and 1% Pen‐Strep until they reached 90% confluency and were changed to FBS‐depleted media, and then the cells were cultured for 48 h. The conditioned medium was filtered with a 0.22 µm filter and stored at −80°C until use. All cells were maintained at 37°C with 5% CO_2_, and passages 3–7 were used for experiments.

### 4.8 VM‐SPARC Formation

Endothelial cells (1 × 10^6^ cells 50 µL^−1^ in PBS) and C2C12 (5 × 10^5^ cells 50 µL^−1^ in PBS) were mixed with blood plasma (1 mL) recalcified with 5 mm CaCl_2._ CaCl_2_ activates endogenous prothrombin in plasma to generate thrombin, which catalyzes the conversion of fibrinogen into fibrin monomers that subsequently assemble into protofibrils and higher‐order fibrin fibers [34]. Subsequently, the blood plasma was introduced into the microfluidic device containing micropillar arrays to fabricate VM‐SPARC. A peristaltic pump (Ismatec Reglo ICC Independent‐Channel Control Peristaltic Pump, ISMATEC) controlled the blood flow rate perfusing through the channel. Blood plasma was perfused at a flow rate of 840 mL h^−1^, gradually reduced to 100 mL h^−1^ while thrombi were developed progressively for 15 min. The VM‐SPARC formed in the device was exposed to air after removing the glass slide. The VM‐SPARC was cultured in the fibroblast‐conditioned medium with 1% horse serum and 1 mg mL^−1^ aminocaproic acid (Sigma‐Aldrich, MA, USA) for 7 days. VM‐SPARC was easily transferred from the microfluidic device using surgical forceps.

### 4.9 Immunocytochemistry

The VM‐SPARC was fixed with 10% formalin and then permeabilized with 0.05% Triton X‐100. Following a wash with PBS, the samples were blocked with 2% BSA buffer for a duration of 1 h. The samples were incubated with primary antibodies in a blocking buffer solution at 4°C overnight. The primary antibody was diluted in 2% BSA buffer at a ratio as follows: anti‐MyoD (Abcam, Cambridge, UK) 1:100, anti‐MyoG (Abcam) 1:100, anti‐MyHC (Abcam) 1:100, anti‐α‐Actinin (Sarcomeric) (Sigma‐Aldrich) 1:500, anti‐CD31 (Thermo Fisher Scientific, MA, USA) 1:100, anti‐Laminin (Abcam) 1:100, anti‐F‐actin (Thermo Fisher Scientific) 1:200, anti‐Ki67 (Abcam) 1:100, and anti‐CD34 (Abcam) 1:100. After washing three times, the samples were incubated with the secondary antibodies diluted in blocking buffer for 2 h at room temperature: goat anti‐rabbit Alexa 488 (Thermo Fisher Scientific), goat anti‐rabbit Alexa 594 (Thermo Fisher Scientific), goat anti‐mouse Alexa 488 (Thermo Fisher Scientific), goat anti‐mouse Alexa 594 (Thermo Fisher Scientific), and Alexa Fluor 488 Anti‐CD31 (Abcam). The engineered thrombi were mounted on glass microscopy slides after staining nuclei with DAPI, and microscopic images were obtained with a confocal microscope (LSM 980, Zeiss, Jena, Germany).

### 4.10 Image Analysis

ImageJ software was used to analyze F‐actin alignment and quantify fluorescence intensity. Cellular alignment was analyzed from F‐actin immunofluorescence images using the OrientationJ plugin. Fluorescence intensities of MyoD, MyoG, MyHC, and SAA were quantified from immunofluorescence images acquired at 20× magnification. For myotube analysis, nuclei were manually counted in MyHC‐positive myotubes longer than 20 µm. The fusion index was calculated as the percentage of nuclei located within multinucleated MyHC‐positive myotubes relative to the total number of nuclei within MyHC‐positive regions. The number of Ki67^+^ lineage‐positive cells per field was manually quantified from immunofluorescence images acquired at 40× magnification. The relative construct area of VM‐Static‐NP and VM‐Static constructs was measured using ImageJ and normalized to the initial construct area at day 0.

### 4.11 qRT‐PCR Analysis

The samples were homogenized in TRIzol (Invitrogen, MA, USA) using a handheld homogenizer. The aqueous phase of the TRIzol extract containing RNA was immediately purified with an RNeasy Mini kit (Qiagen, Hilden, Germany). RNA was quantified by measuring the absorbance at 260 nm using a Synergy Neo2 HTS Multi‐Mode Microplate Reader (BioTek, VT, USA). cDNA was synthesized from 1000 ng of total RNA using a ReverTraAce qPCR RT Master Mix and a gDNA remover kit (Toyobo, Osaka, Japan) following the manufacturer's protocol. qRT‐PCR was performed using a CFX Connect Real‐Time PCR Detection System (Bio‐Rad) with SYBR Green PCR Master Mix (Toyobo). The primer sequences are listed in Table S1. The gene expression was calculated relative to the internal reference genes (*Gapdh* for mouse and *GAPDH* for human) and normalized to the respective control groups: M‐static (Figure 2f), 2D Co‐cultured cells (Figure 3d,e), M‐SPARC (Figure 3h), and V‐SPARC (Figure 3i).

### 4.12 Calcium Imaging

To evaluate intracellular calcium dynamics, samples were incubated with 2 µm Fluo‐4 AM (Thermo Fisher Scientific) for 1 h at 37°C. Excess dye was removed by washing with SPARC basal medium, and fluorescence signals were acquired using a confocal microscope (LSM 710, Carl Zeiss) in time series scanning mode. Regions of interest (ROIs) were defined within individual myotubes, and changes in fluorescence intensity over time were quantified using ZEN software. Calcium transients were monitored before and after stimulation with 200 µm acetylcholine (Thermo Fisher Scientific), and fluorescence changes were expressed as normalized calcium transients (Δ*F*/*F*_0_).

### 4.13 Electrical Stimulation and Functional Assessment

To compare the contractile function of Static‐NP and VM‐SPARC, samples were immersed in Hank's Balanced Salt Solution (HBSS; Thermo Fisher Scientific) pre‐warmed to 37°C. Two sterilized platinum electrodes were placed on either side of the construct and connected to the output channels of a WPG100e electrochemical workstation (WonATech, Seoul, Korea) via electrode cables and alligator clips. Electrical stimulation was applied using biphasic square wave pulses at 10 V with a 50% duty cycle. Bright‐field images of tissue contraction were captured using an Olympus CKX53 microscope (Olympus, Tokyo, Japan). Recorded videos were converted to TIFF image sequences using FFmpeg. The extracted frames were analyzed using the MUSCLEMOTION plugin in ImageJ to quantify contraction parameters, including amplitude [35]. Time‐to‐peak tension (TPT) was defined as the interval between the onset of contraction and the peak amplitude. Half‐relaxation time (HRT) was calculated as the time required for the contraction to decline to 50% of its peak value.

### 4.14 Live/Dead Assay

Cell viability of M‐SPARC, V‐SPARC, and VM‐SPARC was evaluated using a LIVE/DEAD Viability/Cytotoxicity Kit (Thermo Fisher Scientific). The constructs were washed with PBS to minimize background fluorescence and then incubated in PBS containing 2 µm calcein AM and 4 µm ethidium homodimer‐1 (EthD‐1) for 30 min at 37°C under 5% CO_2_. After an additional wash with PBS, live and dead cells were imaged by confocal laser scanning microscopy (LSM 710, Zeiss). Live identified based on calcein AM and EthD‐1 fluorescence, respectively. visualized by confocal laser scanning microscopy (LSM710, Zeiss). Live and dead cells were identified based on calcein AM and EthD‐1 fluorescence, respectively, and manually counted to determine cell viability.

### 4.15 Acellular SPARC Degradation and Cytocompatibility Assay

Acellular SPARC scaffolds were incubated in PBS, high‐glucose DMEM supplemented with 10% FBS, or 0.1 U mL^−1^ plasmin solution (Roche, Switzerland) to evaluate construct degradation. At the indicated time points, constructs were retrieved and weighed, and the relative construct weight was calculated by normalizing to the initial weight at day 0. For cytocompatibility testing, conditioned media collected from the PBS and 10% FBS groups on day 3 and day 7 were mixed at a 1:1 ratio with fresh high‐glucose DMEM and applied to C2C12, HUVEC, and C2C12/HUVEC co‐culture for 48 h. Cell viability was subsequently assessed using a Cell Counting Kit‐8 (CCK‐8; GlpBio, CA, USA) assay.

### 4.16 Subcutaneous Implantation and Biocompatibility Assessment of SPARC Scaffolds

Male 8‐week‐old ICR mice (Orient Bio Inc., Korea) were anesthetized with 1.5% isoflurane, and a dorsal skin incision was made to create a subcutaneous pocket for implantation. The constructs were implanted into the subcutaneous space, and the incision was closed with sutures. Mice subjected to skin incision without scaffold implantation were used as the sham control group. To evaluate systemic toxicity, blood samples were collected by retro‐orbital bleeding before implantation and 1 week after implantation, followed by serum biochemical analysis of aspartate aminotransferase (AST), and alanine aminotransferase (ALT) levels. At 1 week after implantation, the mice were sacrificed, and the implanted tissue was harvested for subsequent histological and immunofluorescence analyses.

### 4.17 Histological and Immunohistochemical Analyses for Biocompatibility Assessment

The harvested tissues were immediately fixed in 10% formalin overnight, embedded in paraffin, sectioned at 6 µm thickness, and deparaffinized through a series of xylene solutions. The sections were then subjected to H&E, MT, TB staining according to standard protocols. Fibrotic area was evaluated on MT‐stained sections by measuring the collagen‐positive area relative to total tissue area within the same field of view. Mast cell density was manually quantified as the number of TB‐positive cells per 1 mm^2^ area.

For immunofluorescence staining, tissue sections were deparaffinized, rehydrated in deionized water, and subjected to antigen retrieval in citrate buffer (Sigma‐Aldrich). After blocking with 2% BSA and 10% goat serum, the sections were incubated overnight at 4°C with primary antibodies diluted in blocking solution as follows: anti‐Ly‐6G/Ly‐6C (Thermo Fisher Scientific) 1:50, anti‐CD86 (Thermo Fisher Scientific) 1:50, and anti‐CD68 (Abcam) 1:200/ The sections were then incubated with the corresponding fluorophore‐conjugated antibodies for 2 h, followed by nuclear staining with DAPI. Fluorescence images were acquired using a confocal laser scanning microscope (LSM 980, Zeiss). The number of Ly‐6G‐positive cells per field and the portion of CD86+/CD68+ macrophages were manually quantified from immunofluorescence images acquired at 40× magnification.

### 4.18 Implantation of VM‐SPARC Into a VML Mouse Model

Male 8‐week‐old SCID nude mice (Orient Bio Inc) were used for animal experiments following the guidelines of the IACUC of UNIST (Approval No. 22–67). Anesthesia of the mice was induced with isoflurane and then maintained with a continuous isoflurane flow. The animals were given a subcutaneous injection of tramadol at a dosage of 20 mg kg^−1^ to control pain. Under sterile conditions, animals were subjected to a muscle defect induced by unilateral resection of the quadratus femoris (QF) muscle using a surgical blade, as previously reported [36, 37]. This defect accounted for a volumetric loss of about 75% of the QF muscle compartment. Immediately afterward, one of the following treatment groups was implanted at the site of the muscle defect: Static, Static‐NP, VM‐SPARC, and Untreated groups. For the transplantation study in the VML model, mice were randomly assigned to Static, Static‐NP, VM‐SPARC, or Untreated groups to undergo histological examination at week 2 (*n* = 4) and week 8 (*n* = 5). Static, Static‐NP, VM‐SPARC, and Untreated groups were observed over a period of 7 weeks for behavioral analysis (*n* = 10)

### 4.19 Histological Analysis for VML Mouse Model

H&E and MT staining standard procedure to examine cross‐sectional size (CSA) and fibrosis. To estimate myofiber CSA, their minimum Feret's diameters were measured using the ImageJ software. Following MT staining, collagen‐content‐based fibrosis was stained blue, and the intensity of this blue dye expression was quantified. Tissue slides were scanned using virtual slide microscopy (Olympus Corporation, Tokyo, Japan), and the blue dye expression was isolated and quantified using the Olympus software for virtual slide microscopy. The procedure allowed for accurate and precise quantification of fibrosis in the tissue samples.

### 4.20 Immunohistochemistry for VML Mouse Model

Immunofluorescence staining was performed as described above. The sections were incubated with primary antibodies: anti‐CD31 (Thermo Fisher Scientific) 1:100, anti‐MyHC (Abcam) 1:100, anti‐MyHC (DSHB) 1:25, anti‐TUBB3 (Cell Signaling Technology, MA, USA) 1:200, anti‐AChR (DSHB) 1:25, anti‐laminin (Abcam) 1:100, DyLight 649‐conjugated Griffonia Simplicifolia Lectin I (GSL I) Isolectin B4 (Vector Laboratories, CA, USA), and fluorescein‐conjugated Ulex Europaeus Agglutinin I (UEA I) (Vector Laboratories). After primary antibody incubation, the sections were treated with the appropriate fluorophore‐conjugated secondary antibodies for 2 h, followed by DAPI staining for nuclear visualization. Images were acquired by a confocal laser microscope (LSM980, Zeiss), where fluorescence signals were measured in an area of 5 mm × 2 mm of the regenerated epithelia. ImageJ was used to quantify the fluorescence intensity of the samples. In addition, AChR‐positive clusters and NMJs were quantified from images acquired at a field size of 210 µm × 210 µm.

### 4.21 Animal Functional Tests

After the surgery, we conducted animal functional tests at 2, 4, 5, 7, and 8 weeks. For the single‐wire hanging test, mice were placed individually at the center of a 40 cm single wire (wire width < 0.2 cm), and hang time was recorded. Each animal had one attempt for 120 s maximally. Physical endurance was evaluated using a treadmill for mice. The mice were kept running on the treadmill at a speed of 15 m min^−1^ and an incline of 20%. When the mice quit running, they were encouraged to run using weak electric stimulation. The test was performed for 60 s, and the number of electric stimulations was recorded.

### 4.22 Statistical Analysis

All data were presented as the mean ± S.E.M. We evaluated the statistical significance of between‐group differences using a two‐tailed Student's *t‐test* or an analysis of variance with Dunnett's post hoc analysis. *p* < 0.05 was considered statistically significant. All statistical analyses were performed using GraphPad Prism (GraphPad Software Inc., CA, USA) and Origin (OriginLab, MA, USA). A sample size (*n*) for each statistical analysis was provided in the figure legend.

## Author Contributions

J.H.K. conceived the study. J.H.K., Y.J., S.H.J., and M.K. designed the experiments. S.H.J. and M.K. fabricated the SPARC using the microfluidic system and performed cellular and molecular analyses. S.H.J. and D.‐Y.K. conducted the in vivo studies, and S.L. performed cytotoxicity experiments. S.H.J. and M.K. analyzed the data, and M.K. developed the theoretical framework describing fibrin bundle formation under shear flow. Y.J. and J.H.K. supervised the project. S.H.J., M.K., Y.J., and J.H.K. wrote the manuscript with input from all authors. All authors reviewed and approved the final manuscript.

## Funding

The National Research Foundation of Korea (NRF) grant funded by the Korea government (Ministry of Science and ICT) (Grant No. RS‐2024‐00344187, RS‐2024‐00399800, RS‐2026‐25469505), the Korean Fund for Regenerative Medicine (KFRM) grant funded by the Korea government (the Ministry of Science and ICT, the Ministry of Health & Welfare) (Grant No. 22A0102L1‐11), the UNIST Research Fund (1.260001.01), the YUCM and YUCD & UNIST Collaborative Project (6‐2024‐0202), and Yonsei University College of Medicine (6‐2021‐0243).

## Conflicts of Interest

The authors declare no conflict of interest.

## Supporting information




**Supporting File**: adma73082‐sup‐0001‐SuppMat.docx.

## Data Availability

The data that support the findings of this study are available in the supplementary material of this article.
