# Supplementary material for: Mechanically Spatio‐Chimeric Fibrin Assembly Enables Vascular‐Integrated Muscle Reconstruction for Volumetric Muscle Loss Repair
Source: Adv Mater. 2026 Apr 22;38(44):e23542. doi: 10.1002/adma.202523542 (PMC13449102; doi:10.1002/adma.202523542)
Supplement: Supplementary file 1 [file ADMA-38-e23542-s001.docx]

Supporting Information

Mechanically Spatio-chimeric Fibrin Assembly Enables Vascular-Integrated Muscle Reconstruction for Volumetric Muscle Loss Repair

Su Hyun Jung^1,†^, Minjun Kim^2,†^, Da-Yoon Kim^1^, Min Kyu Kim^1^, Sieun Lee^2^, Yoonhee Jin^2,*^, and Joo H. Kang^1,*^

^1^Department of Biomedical Engineering, College of Information and Biotechnology, Ulsan National Institute of Science and Technology (UNIST), Ulsan 44919, Republic of Korea

E-mail: [jookang@unist.ac.kr](mailto:jookang@unist.ac.kr)

^2^Department of Physiology, Graduate School of Medical Science, Brain Korea 21 Project, Yonsei University College of Medicine, Seoul 03722, Republic of Korea

E-mail: [yoonheejin@yuhs.ac](mailto:yoonheejin@yuhs.ac)

1. Fibrin bundle formation and its dependence on shear rate

Based on the result of our experiments, we analyzed how shear flow modifies fibrin aggregation kinetics by comparing diffusion- and shear-induced collision rates across relevant length scales ($10 nm-10 \mu m$). Using scaling arguments, we show that the shear rate plays a larger role in fibrin fiber lateral aggregation once the length scale is in the micrometer. A simple scaling argument proceeds as follows: In pioneering work by Smoluchowski^[1]^, the diffusion-induced collision rate of two particles was derived as

$$K_{ij}^{diff}=4\pi\left( D_{i}+D_{j} \right)a_{ij}, (1)$$

where $D_{i}$ and $D_{i}$ are the diffusion coefficients of particles and $a_{c}$ is a capture distance representing the mean distance at which the two particles collide. For two spheres of radius $r_{1}$ and $r_{2}$, $a_{ij}=r_{i}+r_{j}$.

Smoluchowski also found the collision rate due to the shear rate as

$$K_{ij}^{shear}=\frac{4}{3}\dot{\gamma}a_{ij}^{3}, (2)$$

where $\dot{\gamma}$ is the shear rate. Assuming the particles to be spherical, substituting the Stokes-Einstein equation $D_{k}=\frac{k_{B}T}{6\pi\eta r_{k}}$, where $k_{B}$ is the Boltzmann constant, $T$ is the absolute temperature in Kelvin, $\eta$ is the dynamic viscosity in $Pa\cdot s$, and the equation $\left( 1 \right)$ simplifies to

$$K_{ij}^{diff}=\frac{2k_{B}T}{3\eta}\left( \frac{1}{r_{i}}+\frac{1}{r_{j}} \right)\left( r_{i}+r_{j} \right)=\frac{2k_{B}T}{3\eta}\frac{\left( r_{i}+r_{j} \right)^{2}}{r_{i}r_{j}}. (3)$$

Comparing the ratio, we have,

$$\frac{K_{ij}^{shear}}{K_{ij}^{diff}}=\frac{2\dot{\gamma}r_{i}r_{j}\eta\left( r_{i}+r_{j} \right)}{k_{B}T}. (4)$$

Setting $r_{i}=r_{j}=r$, the equation simplifies to

$$\frac{K^{shear}}{K^{diff}}=\frac{4\dot{\gamma}\eta r^{3}}{k_{B}T}, (5)$$

which can be seen as a ratio of viscous dissipation energy (imparted on the sphere) and thermal energy. Using a measured diffusion coefficient of fibrinogen in water of $2.0\times{10}^{-7}cm^{2}s^{-1}$ ^[2]^ and Stokes-Einstein equation at room temperature ($T=298.15 K, \eta=0.85 mPa\cdot s$), the hydrodynamic radius of fibrinogen can be calculated as $r_{H}\approx13 nm$. Substituting this into Eq. (5) with $\eta=4 mPa\cdot s$ (the viscosity of whole blood) and $T=298.15 K$, we obtain

$$\frac{K^{shear}}{K^{diff}}\approx8.54\times{10}^{-6}\cdot\dot{\gamma}$$

Therefore, for collisions between two fibrinogen monomers, even at $\dot{\gamma}\approx1000 s^{-1}$, the shear rate is inconsequential.

However, it is known that the shear rate does influence the polymerization kinetics of protofibrils^[3]^. This becomes evident upon substituting in $r_{i}=1 \mu m$ and $r_{j}=10 nm$ into equation (4). With the other parameter values fixed as before, we obtain

$$\frac{K^{shear}}{K^{diff}}\approx0.02\cdot\dot{\gamma},$$

which shows that even at moderate shear rate ($\dot{\gamma}\approx15 s^{-1})$, the shear flow can indeed affect the kinetics of protofibril polymerization at the micrometer scale, as seen in the work of Tippe and Muller-Mohnssen^[3]^. For fibrin fiber-fiber bundle formation, with a length scale at micrometer, our experiments indicate that shear flow is the dominant mechanism, while diffusion plays only a minor role. This is evident when substituting $r=0.5 \mu m$ into Eq. (5), which yields

$$\frac{K^{shear}}{K^{diff}}\approx0.49\cdot\dot{\gamma}.$$

Thus, as the fibrin fiber size reaches a micrometer scale, the shear rate becomes a significant factor even at a modest value. Since it is known that the lateral bundling of fibrin fiber is generally followed by fibrin polymerization^[4-6]^, this argument is in accordance with our experimental results.

While the preceding argument is valid only for spheres, substituting the hydrodynamic radius of fibrinogen or fibrin fibers yields an order of magnitude threshold at which collision due to advection exceeds that of thermal diffusion. A stricter, yet approximate argument can be made based on the work of Tirado and Garcia de la Torre, who had worked on the diffusion of cylindrical molecules, such as DNA^[7]^. Based on their work, the translational diffusion coefficient $D_{t}$ for a slender rod is given as

$$D_{t}=\frac{1}{3}\left( D_{\parallel}+2D_{\perp} \right)$$

where $D_{\parallel}, D_{\perp}$ are axial and transversal diffusion coefficient of a rod, respectively, and $L$ is the length of the cylinder. The formulas for $D_{\parallel}, D_{\perp}$ are given as^[7-10]^

$$D_{\parallel}=\frac{k_{B}T}{2\pi\eta L}\left( \ln\frac{L}{d}+\nu_{\parallel} \right), D_{\perp}=\frac{k_{B}T}{4\pi\eta L}\left( \ln\frac{L}{d}+\nu_{\perp} \right),$$

where $d$ is the diameter of the cylinder and $\nu_{\parallel}$ and $\nu_{\perp}$ are small correction factors that are less than 1 in magnitude. Ignoring the correction factors, the effective diffusion equation is given by

$$D_{t}=\frac{1}{3}\left( D_{\parallel}+2D_{\perp} \right)\approx\frac{k_{B}T}{3\pi\eta L}\ln\frac{L}{d}.$$

A crucial assumption here that deviates from^[11]^ is that the fibrin fibers are mostly aligned with the shear flow—that orientation is not random—as shown in Figure 1. In such a case, for identical cylinders, the equation (1) reduces to $K^{diff}\approx8\pi D_{eff}d$, and we obtain

$$K^{diff}\approx\frac{8k_{B}T}{3\eta}\frac{d}{L}\ln\frac{L}{d}. \left( 6 \right)$$

It should be noted that $K^{diff}\propto L^{-1}$ and that this is due to the alignment of fibrin fiber with the shear flow (For random orientation, as assumed in^[12]^, $a_{c}\approx L$ so that $K^{diff}$ is independent of particle size). Lastly, this formula is valid only for $L/d\gg1$.

To the best of our knowledge, no closed form has been derived for the shear-induced collision rate for a cylinder. However, based on the work of^[11]^, for high aspect ratio ($L/d\gg1$), the ideal collision rate of a cylinder is $O\left( Ld^{2}\ln\frac{L}{d} \right)$ so that we may write

$$K^{shear}\approx\chi\frac{4}{3}\dot{\gamma}Ld^{2}\ln\frac{L}{d}, (7)$$

where $\chi=O\left( 1 \right)$ collects orientation statistics (Jeffery orbits) and hydrodynamic details. $\chi\approx1$ in dilute, weakly aligned suspension and decreases with alignment. Here, we set $\chi=1$ for convenience.

Comparing the ratio, we have

$$\frac{K^{shear}}{K^{diff}}\approx\frac{\dot{\gamma}\eta L^{2}d}{2k_{B}T}. (8)$$

It is interesting to note that we recover Eq. (5) as $L\to d$. Substituting in $L=6 \mu m, d=120 nm$—which has sphere-equivalent radius of roughly $0.5 \mu m$ and an aspect ratio of 50, a modest value for fibrin fiber—yields

$$\frac{K^{shear}}{K^{diff}}\approx2.1\dot{\gamma.}$$

Now, the smallest fibrin fiber bundle we found in the high shear region was around $d=8 \mu m$ (Figure 1f), and using a lower bound aspect ratio of 10 ($L=80 \mu m$), we get

$$\frac{K^{shear}}{K^{diff}}\approx2.5\times{10}^{4}\dot{\gamma},$$

which indicates that at the scale of our interest, which is in $1\sim100 \mu m$, the collision due to diffusion is essentially negligible.

The above argument gives a theoretical foundation as to why shear rate is the critical factor in fibrin fiber bundling as opposed to that of diffusion. A few remarks are in order here. First, collision does not equate to polymerization or bundling. For instance, the kinetic ODE model of fibrin polymerization shows that the kinetic rate is orders of magnitude smaller than that of the diffusion-limited kinetic constant, indicating that only a fraction of collisions result in polymerization. The same most likely holds for fibrin bundling, where adhesive force between fibers must overcome the shear stress. A simple argument can show that if the area of an attractive site is $\sigma^{2}$, then the probability of two fibers sticking together is on the order of $\left( {\sigma^{2}}/{\pi dL} \right)^{2}$. From this, the shortcoming of the above argument is evident in that inter-particle forces and hydrodynamic interactions are neglected^[13-16]^. Secondly, one should not jump to the conclusion that $\frac{K^{shear}}{K^{diff}}=O(\dot{\gamma}L^{2}d)$. This was derived from the assumption that fibrin fibers are aligned with the shear flow and in fact, for randomly oriented fibers, $\frac{K^{shear}}{K^{diff}}=O(\dot{\gamma}L^{3}) ADDIN ZOTERO\_ITEM CSL\_CITATION \{"citationID":"dmexfQwZ","properties":\{"formattedCitation":"\backslash\backslash super [51]\backslash\backslash nosupersub\{\}","plainCitation":"[51]","noteIndex":0\},"citationItems":[\{"id":1118,"uris":["http://zotero.org/users/7522981/items/SS9T34BL"],"itemData":\{"id":1118,"type":"article-journal","abstract":"The collision of particles influences the behavior of suspensions through the formation of aggregates for adhesive particles or through the contributions of solid-body contacts to the stress for nonadhesive particles. The simplest estimate of the collision rate, termed the ideal collision rate, is obtained when particles translate and rotate with the flow but have no hydrodynamic or colloidal interactions. Smoluchowski calculated the ideal collision frequency of spherical particles in 1917. So far, little work has been done to understand rate of collision for nonspherical particles. In this work, we calculate the ideal collision rate for cylindrical particles over a broad range of particle aspect ratios r defined as the ratio of length to diameter. Monte Carlo simulations are performed with initial relative positions and orientations that model the rate of approach of noninteracting particles following Jeffery orbits with several choices of the orbit distribution. The role of rotational motion of particles on collision frequency is elucidated by comparing the ideal collision rate calculations with similar calculations for nonrotating particles. It is shown that the ratio of the collision rate of cylinders to that of spheres that circumscribe the cylinders is proportional to 1/rre for r \gg1 and re for r \ll1. Here, re is the effective aspect ratio defined as the aspect ratio of a spheroid having the same period of rotation as the cylinder. The effective aspect ratio of the cylindrical particles was determined using finite element calculations of the torque on nonrotating cylinders with their axes parallel to the velocity and velocity gradient directions. In addition to deriving the total collision rate, we categorize collisions as side-side, edge-side, and face-edge based on the initial point of contact. Most collisions are found to be side-edge for r \gg1 and face-edge for r \ll1, suggesting that nonlinear aggregates will develop if particles stick at the point of first contact.","container-title":"Langmuir","DOI":"10.1021/la202138t","ISSN":"0743-7463","issue":"19","journalAbbreviation":"Langmuir","page":"11813-11823","publisher":"American Chemical Society","source":"ACS Publications","title":"Ideal Rate of Collision of Cylinders in Simple Shear Flow","URL":"https://doi.org/10.1021/la202138t","volume":"27","author":[\{"family":"Singh","given":"Vikram"\},\{"family":"Koch","given":"Donald L."\},\{"family":"Stroock","given":"Abraham D."\}],"accessed":\{"date-parts":[["2025",11,22]]\},"issued":\{"date-parts":[["2011",10,4]]\}\}\}],"schema":"https://github.com/citation-style-language/schema/raw/master/csl-citation.json"\}$^[11]^. Since larger fibers are more easily aligned by the shear flow, the scaling law most likely depends on the size and the geometry of the system.

In any case, our experiments have shown that shear rate plays a significant role in fibrin fiber bundling, and it can be extrapolated from the previous argument that as fibrin fiber diameter reaches the micrometer scale, diffusion alone is not sufficient to explain the large bundles we have observed in our experiments.

**Supplementary References**

1.	M. Smoluchowski, "Versuch einer mathematischen Theorie der Koagulationskinetik kolloider Lösungen" *Zeitschrift für Physikalische Chemie* (1918**)**, *92U*, 129.

2.	G. Truskey, F. Yuan, D. Katz, in *Pearson Prentice Hall, Upper Saddle River, NJ*, (2009), p.888.

3.	A. Tippe, H. Müller-Mohnssen, "Shear dependence of the fibrin coagulation kinetics in vitro" *Thrombosis Research* (1993**)**, *72*, 379.

4.	R. R. Hantgan, J. Hermans, "Assembly of fibrin. A light scattering study" *Journal of Biological Chemistry* (1979**)**, *254*, 11272.

5.	J. W. Weisel, "Fibrin assembly. Lateral aggregation and the role of the two pairs of fibrinopeptides" *Biophysical Journal* (1986**)**, *50*, 1079.

6.	J. W. Weisel, R. I. Litvinov, "Mechanisms of fibrin polymerization and clinical implications" *Blood* (2013**)**, *121*, 1712.

7.	S. Broersma, "Rotational Diffusion Constant of a Cylindrical Particle" *The Journal of Chemical Physics* (1960**)**, *32*, 1626.

8.	M. M. Tirado, J. G. Torre, "Translational friction coefficients of rigid, symmetric top macromolec ules. Application to circular cylinders" *The Journal of Chemical Physics* (1979**)**, *71*, 2581.

9.	M. M. Tirado, J. G. Torre, "Rotational dynamics of rigid, symmetric top macromolecules. Application to circular cylinders" *The Journal of Chemical Physics* (1980**)**, *73*, 1986.

10.	M. M. Tirado, C. L. Martínez, J. G. Torre, "Comparison of theories for the translational and rotational diffusion coefficients of rod‐like macromolecules. Application to short DNA fragments" *The Journal of Chemical Physics* (1984**)**, *81*, 2047.

11.	V. Singh, D. L. Koch, A. D. Stroock, "Ideal Rate of Collision of Cylinders in Simple Shear Flow" *Langmuir* (2011**)**, *27*, 11813.

12.	A. P. Philipse, A. M. Wierenga, "On the Density and Structure Formation in Gels and Clusters of Colloidal Rods and Fibers" *Langmuir* (1998**)**, *14*, 49.

13.	D. L. Swift, S. K. Friedlander, "The coagulation of hydrosols by brownian motion and laminar shear flow" *Journal of Colloid Science* (1964**)**, *19*, 621.

14.	D. L. Feke, W. R. Schowalter, "The influence of brownian diffusion on binary flow-induced collision rates in colloidal dispersions" *Journal of Colloid and Interface Science* (1985**)**, *106*, 203.

15.	D. L. Feke, W. R. Schowalter, "The effect of Brownian diffusion on shear-induced coagulation of colloidal dispersions" *Journal of Fluid Mechanics* (1983**)**, *133*, 17.

16.	D. N. Thomas, S. J. Judd, N. Fawcett, "Flocculation modelling: a review" *Water Research* (1999**)**, *33*, 1579.

**Figure S1.** **Quantitative analysis of flow rate–dependent fibrin fiber alignment within the microfluidic device.** Representative SEM images and corresponding orientation index analysis illustrate the progressive alignment of fibrin fibers under increasing flow rates (0, 10, 200, and 840 mL h⁻¹).

**Figure S2. A schematic illustration depicting shear-dominated fibrin bundling across scales under increasing flow shear rates.** (Left) Under static or diffusion-dominated conditions, fibrin monomers interact through random Brownian collisions, forming protofibrils and thin fibers with limited lateral association. (Right) As shear rates increase, hydrodynamic forces drive frequent, directed collisions among fibrin fibers and protofibrils, promoting lateral aggregation and the formation of thicker fibrin bundles. This shear-enhanced bundling represents a transition from diffusion-controlled polymerization at the nanoscale to shear-driven assembly at the microscale.

**Figure S3. Fibrin fiber morphology and alignment at different NaCl concentrations.** SEM images showing fibrin fiber structures formed at NaCl concentrations of 0, 50, and 100 mM. Quantitative analyses present changes in fiber bundle size and alignment under each condition.

**Figure S4. Shear-induced fibrin alignment in myoblast-containing constructs.** Representative SEM images of fibrin networks in M-Static, M-Static-NP, and M-SPARC immediately after assembly (day 0). Myoblasts are pseudo-colored in magenta for visualization. Corresponding quantitative fiber orientation distributions are shown beside each image. Scale bars = 10–20 μm.

**Figure S5. Severe shrinkage of VM-Static-NP constructs during *in vitro* culture.** a) Representative images of VM-Static-NP and VM-Static constructs over time (D0–D7), showing pronounced contraction and reduction in construct size in VM-Static-NP, whereas VM-Static maintains its original geometry. Scale bars = 1 cm. b) Quantification of the relative construct area over time normalized to day 0. VM-Static-NP constructs progressively decreased to approximately 60.78% at day 3 and 12.53% at day 7, while VM-Static constructs maintained approximately 96.31% of their initial area during the same period. (*n* = 5).

**Figure S6.** **Endothelial network formation within SPARC.** Representative immunofluorescence images of CD31 (green) and CD34 (red) staining showing endothelial organization within VM-Static (with no shear) and VM-SPARC. CD34-positive structures were observed predominantly in the low-shear region. Nuclei are stained with DAPI (blue). Scale bars = 50 μm.

**Figure S7. Shear-dependent proliferation of endothelial and myogenic cells in VM-SPARC.** Representative immunofluorescence images showing Ki67 staining with lineage-specific markers in VM-Static and VM-SPARC. Endothelial cells are identified by CD31 and myogenic cells by sarcomeric α-actinin (SAA). Individual fluorescence channels (SAA, CD31, and DAPI) are shown separately for clarity. Images were obtained from VM-Static and from low- and high-shear regions of VM-SPARC. Scale bars = 50 μm.

**Figure S8. Cytocompatibility evaluation of SPARC constructs**. a) Representative Live/Dead staining images of shear-assembled muscle constructs (M-SPARC), shear-assembled vascularized constructs (V-SPARC), and vascularized muscle constructs (VM-SPARC) at day 3 of culture. For VM-SPARC, images were obtained from regions corresponding to low shear (γ̇ < 100 s⁻¹) and high shear (γ̇ > 100 s⁻¹) regimes. Live cells are shown in green and dead cells in red. Scale bars = 100 μm. b) Quantification of cell viability across the different construct types and shear regions. No significant differences in cell viability were observed among group.

**Figure S9. Structural stability of the SPARC architecture during extended *in vitro* culture.** a, b) Quantitative analysis of fibrin bundle diameter and orientation in VM-Static and VM-SPARC after 7 days of *in vitro* culture. Frequency distribution of fibrin bundle diameter and corresponding orientation plots for the regions 1-4 analyzed in the SEM images of VM-Static (a) and VM-SPARC (b) constructs shown in Figure 3j. c) SEM images of VM-SPARC after 14 days of *in vitro* culture. The left image shows a low-magnification overview of the construct (scale bar = 100 μm). The four images on the right show higher-magnification SEM views of the construct (scale bars = 20 μm and 50 μm), with corresponding orientation plots indicating the overall structural alignment.

**Figure S10. Degradation behavior and cytocompatibility of SPARC scaffolds.** a) Representative images of cell-free SPARC scaffolds incubated under different degradation conditions, including PBS, serum-containing medium (10% FBS), and plasmin solution (0.1 U mL⁻¹), at indicated time points (Day 0~Day 7). b) Quantification of scaffold degradation expressed as relative weight (%) over time under the three conditions. c) Cytocompatibility of degradation products evaluated using conditioned media (CM) collected at day 3 and day 7 from PBS and 10% FBS degradation conditions. Cell viability of C2C12-HUVEC co-cultures was assessed by CCK-8 assays after 48 h exposure to CM (50% v/v).

**Figure S11. *In vivo* biocompatibility and immunogenicity evaluation of SPARC scaffolds.** a) Representative Hematoxylin and Eosin (H&E), Masson's trichrome (MT), and Toluidine blue (TB) staining of Sham and Implant groups at 1 week after subcutaneous implantation. Scale bars = 200 μm. b) Quantification of fibrotic area based on MT staining (*n* = 6-8). c) Quantification of mast cell numbers per tissue area based on TB staining (*n* = 8). d) Representative immunofluorescence staining for Ly-6G (green) and nuclei (DAPI, blue) showing neutrophil infiltration in Sham and Implant groups. Scale bars = 50 μm. e) Quantification of Ly-6G⁺ cells per field (*n* = 5-6). f) Representative immunofluorescence staining for CD86 (green), CD68 (red), and nuclei (DAPI, blue) showing M1 macrophages. Scale bars = 50 μm. g) Quantification of CD86⁺/CD68⁺ macrophage proportion (*n* = 5). h) Serum biochemical analysis of AST and ALT levels in Sham and Implant groups for systemic toxicity evaluation (*n* = 4). n.s., not significant. Statistical analysis was performed using one-way ANOVA followed by Tukey’s post hoc test (*P < 0.05, **P < 0.01, ***P < 0.001).

**Supplementary Table S1. Primer sequences used in this study**

| Gene | Forward Primer (5’ - 3’) | Reverse Primer (5’ - 3’) |
| --- | --- | --- |
| *Gapdh* (MOUSE) | AACACAGTCCATGCCATCAC | TCCACCACCCTGTTGCTGTA |
| *GAPDH* (HUMAN) | GACAGTCAGCCGCATCTTCT | TAGCCTCCCGGGTTTCTCTC |
| Myod1 | CCACTCCGGGACATAGACTTG | AAAAGCGCAGGTCTGGTGAG |
| Myf5 | AAGGCTCCTGTATCCCCTCAC | TGACCTTCTTCAGGCGTCTAC |
| Myog | GCAGGCTCAAGAAAGTGAATGA | TAGGCGCTCAATGTACTGGAT |
| Myh1 | CTCTTCCCGCTTTGGTAAGTT | CAGGAGCATTTCGATTAGATCCG |
| Myh2 | GCACCCATCCTCATTTCGTGA | GGAATGGCACTTGCGTTTAACA |
| Myh4 | CTTTGCTTACGTCAGTCAAGGT | AGCGCCTGTGAGCTTGTAAA |
| Myh7 | AGACTGTCAACACTAAGAGGGT | TGCCCCAAAATGGATTCGGAT |
| *Mstn* | AACCTTCCCAGGACCAGGAGAA | GGCTTCAAAATCGACCGTGAGG |
| *Fbxo32* | CTTCTCGACTGCCATCCTGGAT | TCTTTTGGGCGATGCCACTCAG |
| *Trim63* | TACCAAGCCTGTGGTCATCCTG | ACGGAAACGACCTCCAGACATG |
| *Ppargc1a* | GAATCAAGCCACTACAGACACCG | CATCCCTCTTGAGCCTTTCGTG |
| *Bcl2* | CCTGTGGATGACTGAGTACCTG | AGCCAGGAGAAATCAAACAGAGG |
| *Bax* | AGGATGCGTCCACCAAGAAGCT | TCCGTGTCCACGTCAGCAATCA |
| CD31 | TCTATGACCTCGCCCTCCACAAA | GAACGGTGTCTTCAGGTTGGTATTTCA |
| CD34 | AAATCCTCTTCCTCTGAGGCTGGA | AAGAGGCAGCTGGTGATAAGGGTT |
| *CD144* | AGCCCAAAGTGTGTGAGAACGC | CTGAGATGACCACGGGTAGGAA |
| VWF | TTCCAGAATGGCAAGAGAGTG | TGAGTTGGCAAAGTCATAAGG |
| *TIE2* | GGTCAAGCAACCCAGCCTTTTC | CAGGTCATTCCAGCAGAGCCAA |
